# 2-(4-Fluoro­phen­yl)-3-hy­droxy-4*H*-chromen-4-one

**DOI:** 10.1107/S1600536810045083

**Published:** 2010-11-10

**Authors:** Michał Wera, Ilia E. Serdiuk, Alexander D. Roshal, Jerzy Błażejowski

**Affiliations:** a Faculty of Chemistry, University of Gdańsk, J. Sobieskiego 18, 80-952 Gdańsk, Poland; b Institute of Chemistry, V.N. Karazin National University, Svobody 4, 61077 Kharkiv, Ukraine

## Abstract

In the crystal structure of the title compound, C_15_H_9_FO_3_, inversely oriented mol­ecules form inversion dimers through pairs of O—H⋯O hydrogen bonds. The benzene ring is twisted at an angle of 12.0 (1)° relative to the 4*H*-chromene skeleton of the mol­ecule. Adjacent 4*H*-chromene units are parallel in a given column or oriented at an angle of 50.0 (1)° in neighboring, inversely oriented, columns, forming a herringbone pattern.

## Related literature

For general background to fluorescence in flavanol (3-hy­droxy-2-phenyl-4*H*-chromen-4-one) and its derivatives, see: Demchenko *et al.* (2002 ▶); Pivovarenko *et al.* (2005 ▶); Roshal *et al.* (2003 ▶); Sengupta & Kasha (1979 ▶). For related structures, see: Cantrell & Stalzer (1982 ▶); Etter *et al.* (1986 ▶); Waller *et al.* (2003 ▶). For inter­molecular inter­actions, see: Aakeröy *et al.* (1992 ▶); Dorn *et al.* (2005 ▶). For the synthesis, see: Smith *et al.* (1968 ▶).
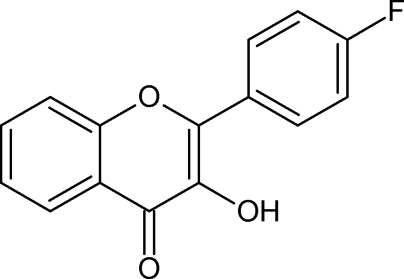


## Experimental

### 

#### Crystal data

C_15_H_9_FO_3_
*M* *_r_* = 256.22
Monoclinic, 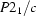

*a* = 15.5971 (9) Å
*b* = 3.8790 (2) Å
*c* = 19.1655 (12) Å
β = 103.906 (6)°
*V* = 1125.55 (11) Å^3^
*Z* = 4
Mo *K*α radiation
μ = 0.12 mm^−1^
*T* = 295 K
0.6 × 0.4 × 0.05 mm

#### Data collection

Oxford Diffraction Gemini R Ultra Ruby CCD diffractometer
Absorption correction: multi-scan (*CrysAlis RED*; Oxford Diffraction, 2008 ▶) *T*_min_ = 0.956, *T*_max_ = 0.991
7973 measured reflections
1999 independent reflections
1729 reflections with *I* > 2σ(*I*)
*R*_int_ = 0.026

#### Refinement

*R*[*F* ^2^ > 2σ(*F* ^2^)] = 0.050
*wR*(*F* ^2^) = 0.113
*S* = 1.12
1999 reflections
176 parameters
H atoms treated by a mixture of independent and constrained refinement
Δρ_max_ = 0.21 e Å^−3^
Δρ_min_ = −0.21 e Å^−3^

### 

Data collection: *CrysAlis CCD* (Oxford Diffraction, 2008 ▶); cell refinement: *CrysAlis RED* (Oxford Diffraction, 2008 ▶); data reduction: *CrysAlis RED*; program(s) used to solve structure: *SHELXS97* (Sheldrick, 2008 ▶); program(s) used to refine structure: *SHELXL97* (Sheldrick, 2008 ▶); molecular graphics: *ORTEP-3* (Farrugia, 1997 ▶); software used to prepare material for publication: *SHELXL97* and *PLATON* (Spek, 2009 ▶).

## Supplementary Material

Crystal structure: contains datablocks global, I. DOI: 10.1107/S1600536810045083/xu5076sup1.cif

Structure factors: contains datablocks I. DOI: 10.1107/S1600536810045083/xu5076Isup2.hkl

Additional supplementary materials:  crystallographic information; 3D view; checkCIF report

## Figures and Tables

**Table 1:** Hydrogen-bond geometry (Å, °)

*D*—H⋯*A*	*D*—H	H⋯*A*	*D*⋯*A*	*D*—H⋯*A*
O11—H11⋯O12	0.83 (3)	2.28 (3)	2.722 (2)	113 (3)
O11—H11⋯O12^i^	0.83 (3)	2.02 (3)	2.761 (2)	149 (3)

Symmetry code: (i) 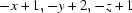.

**Fig. 1.**
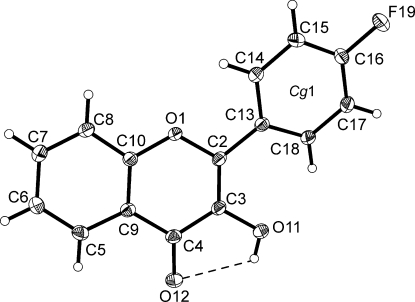
The molecular structure of the title compound showing the atom labeling scheme. Displacement ellipsoids are drawn at the 25% probability level, and H atoms are shown as small spheres of arbitrary radius. The O—H···O hydrogen bond is indicated by a dashed line.

**Fig. 2.**
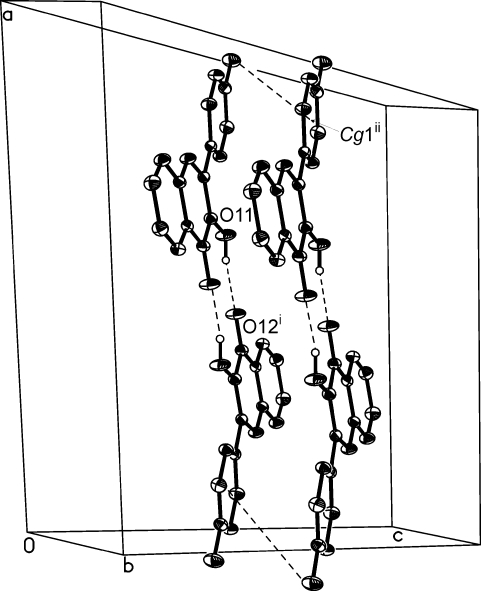
The arrangement of the molecules in the crystal structure. The O—H···O hydrogen bonds are represented by dashed lines, the C—F···π contacts by dotted lines. H atoms not involved in interactions have been omitted. [Symmetry codes: (i) –x + 1, –y + 2, –z + 1; (ii) x, y – 1, z.]

**Fig. 3.**
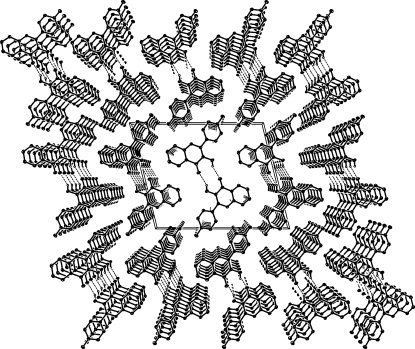
Columns in the crystal structure, viewed along the b axis. The O—H···O interactions are represented by dashed lines, the C—F···π contacts by dotted lines. H atoms not involved in interactions have been omitted.

## supplementary crystallographic information

### Comment

3-Hydroxy-2-phenyl-4*H*-chromen-4-one (flavonol) and its derivatives exhibit dual fluorescence in liquid phases originating from the Excited State Intramolecular Proton Transfer (ESIPT) phenomenon (Sengupta & Kasha, 1979). Since the fluorescence of flavonols depends strongly on the properties of the medium, the compounds can be applied as analytical probes in chemistry, biochemistry, biology and medicine (Demchenko *et al.*, 2002). Continuing our investigations into this group of compounds (Roshal *et al.*, 2003; Pivovarenko *et al.*, 2005) we now present the crystal structure of a flavonol derivative – 2-(4-fluorophenyl)-3-hydroxy-4*H*-chromen-4-one.

In the title compound (Fig. 1), the bond lengths and angles characterizing the geometry of the 2-phenyl-4*H*-chromen-4-one (flavone) moiety are typical of this group of compounds (Cantrell & Stalzer, 1982; Etter *et al.*, 1986; Waller *et al.*, 2003). With respective average deviations from planarity of 0.0147 (2)° and 0.0020 (2)°, the 4*H*-chromene and benzene ring systems are oriented at a dihedral angle of 12.0 (1)° (in the case of flavonol this angle is equal to 5.5 (1)° (Etter *et al.*, 1986)).

In the crystal structure, the inversely oriented molecules form dimers through a pair of intermolecular O—H···O (Aakeröy *et al.*, 1992) bonds (Table 1, Fig. 2). Dimers oriented in parallel – linked by C—F···π (Dorn *et al.*, 2005) contacts (Table 2, Fig. 2) – are arranged in columns along the *b* axis which are dispersively stabilized in the crystal lattice. The adjacent 4*H*-chromene moieties are parallel in a given column or oriented at an angle of 50.0 (1) in the two neighboring, inversely oriented, columns, which forms a herringbone pattern. The O11—H11···O12 intramolecular hydrogen bond (Table 1, Figs. 1 and 2) is the one involved in the ESIPT phenomenon, characteristic of flavonols (Sengupta & Kasha, 1979).

### Experimental

The title compound was synthesized following the procedure described by Smith *et al.*, 1968. Briefly, 3-(4-fluorophenyl)-1-(2-hydroxyphenyl)prop-2-en-1-one was synthesized first by the condensation of 1-(2-hydroxyphenyl)ethanone with 4-fluorobenzaldehyde in methanol/50% aqueous NaOH (1/1 *v*/*v*), precipitated by neutralizing the reaction mixture with aqueous HCl and separated by filtration. The product thus obtained was subjected to oxidative cyclization in alkaline methanol/H_2_O_2_ to yield 2-(4-fluorophenyl)-3-hydroxy-4*H*-chromen-4-one. The filtered product was purified chromatographically (Silica Gel, chloroform/methanol, 20/1 *v*/*v*) and yellow crystals suitable for X-ray investigations were grown from absolute ethanol (m.p. = 442–443 K).

### Refinement

The H atoms of the C—H bonds were positioned geometrically, with C—H = 0.93 Å, and constrained to ride on their parent atoms with *U*_iso_(H) = 1.2*U*_eq_(C). The H atoms involved in O—H···O hydrogen bonds were located on a difference map and refined freely with *U*_iso_(H) = 1.2*U*_eq_(O).

### Crystal data

**Table:**

C_15_H_9_FO_3_	*F*(000) = 528
*M**_r_* = 256.22	*D*_x_ = 1.512 Mg m^−^^3^
Monoclinic, *P*2_1_/*c*	Mo *K*α radiation, λ = 0.71073 Å
Hall symbol: -P 2ybc	Cell parameters from 1729 reflections
*a* = 15.5971 (9) Å	θ = 3.9–25.1°
*b* = 3.8790 (2) Å	µ = 0.12 mm^−^^1^
*c* = 19.1655 (12) Å	*T* = 295 K
β = 103.906 (6)°	Plate, yellow
*V* = 1125.55 (11) Å^3^	0.6 × 0.4 × 0.05 mm
*Z* = 4	

### Data collection

**Table:**

Oxford Diffraction Gemini R Ultra Ruby CCD diffractometer	1999 independent reflections
Radiation source: Enhance (Mo) X-ray Source	1729 reflections with *I* > 2σ(*I*)
graphite	*R*_int_ = 0.026
Detector resolution: 10.4002 pixels mm^-1^	θ_max_ = 25.1°, θ_min_ = 3.9°
ω scans	*h* = −18→14
Absorption correction: multi-scan (*CrysAlis RED*; Oxford Diffraction, 2008)	*k* = −4→4
*T*_min_ = 0.956, *T*_max_ = 0.991	*l* = −20→22
7973 measured reflections	

### Refinement

**Table:**

Refinement on *F*^2^	Secondary atom site location: difference Fourier map
Least-squares matrix: full	Hydrogen site location: inferred from neighbouring sites
*R*[*F*^2^ > 2σ(*F*^2^)] = 0.050	H atoms treated by a mixture of independent and constrained refinement
*wR*(*F*^2^) = 0.113	*w* = 1/[σ^2^(*F*_o_^2^) + (0.0392*P*)^2^ + 0.833*P*] where *P* = (*F*_o_^2^ + 2*F*_c_^2^)/3
*S* = 1.12	(Δ/σ)_max_ < 0.001
1999 reflections	Δρ_max_ = 0.21 e Å^−^^3^
176 parameters	Δρ_min_ = −0.21 e Å^−^^3^
0 restraints	Extinction correction: *SHELXL97* (Sheldrick, 2008), Fc^*^=kFc[1+0.001xFc^2^λ^3^/sin(2θ)]^-1/4^
Primary atom site location: structure-invariant direct methods	Extinction coefficient: 0.010 (2)

### Special details

**Table:**

Geometry. All e.s.d.'s (except the e.s.d. in the dihedral angle between two l.s. planes) are estimated using the full covariance matrix. The cell e.s.d.'s are taken into account individually in the estimation of e.s.d.'s in distances, angles and torsion angles; correlations between e.s.d.'s in cell parameters are only used when they are defined by crystal symmetry. An approximate (isotropic) treatment of cell e.s.d.'s is used for estimating e.s.d.'s involving l.s. planes.
Refinement. Refinement of *F*^2^ against ALL reflections. The weighted *R*-factor *wR* and goodness of fit *S* are based on *F*^2^, conventional *R*-factors *R* are based on *F*, with *F* set to zero for negative *F*^2^. The threshold expression of *F*^2^ > σ(*F*^2^) is used only for calculating *R*-factors(gt) *etc*. and is not relevant to the choice of reflections for refinement. *R*-factors based on *F*^2^ are statistically about twice as large as those based on *F*, and *R*- factors based on ALL data will be even larger.

### Fractional atomic coordinates and isotropic or equivalent isotropic displacement parameters (Å^2^)

**Table:**

	*x*	*y*	*z*	*U*_iso_*/*U*_eq_	
O1	0.78326 (8)	0.9690 (4)	0.42669 (7)	0.0375 (4)	
C2	0.75381 (12)	0.8861 (5)	0.48666 (10)	0.0305 (5)	
C3	0.66843 (13)	0.9437 (6)	0.48778 (10)	0.0340 (5)	
C4	0.60607 (13)	1.0994 (6)	0.42768 (11)	0.0350 (5)	
C5	0.58939 (13)	1.3469 (6)	0.30419 (11)	0.0376 (5)	
H5	0.5310	1.4047	0.3022	0.045*	
C6	0.62406 (14)	1.4144 (6)	0.24658 (12)	0.0421 (6)	
H6	0.5891	1.5134	0.2052	0.051*	
C7	0.71196 (15)	1.3346 (7)	0.25000 (12)	0.0442 (6)	
H7	0.7354	1.3816	0.2108	0.053*	
C8	0.76441 (14)	1.1878 (7)	0.31031 (11)	0.0423 (6)	
H8	0.8232	1.1364	0.3124	0.051*	
C9	0.64095 (12)	1.1912 (5)	0.36633 (10)	0.0306 (5)	
C10	0.72829 (12)	1.1169 (6)	0.36824 (10)	0.0320 (5)	
O11	0.63946 (10)	0.8624 (5)	0.54678 (8)	0.0542 (5)	
H11	0.585 (2)	0.891 (9)	0.5380 (16)	0.081*	
O12	0.52826 (9)	1.1478 (5)	0.42954 (9)	0.0554 (5)	
C13	0.82586 (12)	0.7435 (5)	0.54311 (10)	0.0306 (5)	
C14	0.91251 (13)	0.7678 (6)	0.53545 (11)	0.0389 (5)	
H14	0.9230	0.8692	0.4943	0.047*	
C15	0.98280 (14)	0.6447 (6)	0.58754 (11)	0.0420 (6)	
H15	1.0402	0.6623	0.5818	0.050*	
C16	0.96660 (13)	0.4969 (6)	0.64746 (11)	0.0369 (5)	
C17	0.88298 (14)	0.4651 (6)	0.65736 (11)	0.0406 (6)	
H17	0.8736	0.3620	0.6987	0.049*	
C18	0.81276 (13)	0.5880 (6)	0.60519 (11)	0.0366 (5)	
H18	0.7557	0.5667	0.6116	0.044*	
F19	1.03595 (8)	0.3745 (4)	0.69849 (7)	0.0561 (4)	

### Atomic displacement parameters (Å^2^)

**Table:**

	*U*^11^	*U*^22^	*U*^33^	*U*^12^	*U*^13^	*U*^23^
O1	0.0255 (7)	0.0598 (10)	0.0287 (7)	0.0049 (7)	0.0097 (6)	0.0056 (7)
C2	0.0284 (10)	0.0368 (12)	0.0278 (10)	−0.0038 (9)	0.0098 (8)	−0.0027 (9)
C3	0.0294 (10)	0.0435 (12)	0.0310 (10)	−0.0026 (10)	0.0109 (8)	−0.0009 (10)
C4	0.0262 (10)	0.0423 (12)	0.0375 (11)	−0.0016 (9)	0.0095 (9)	−0.0022 (10)
C5	0.0281 (10)	0.0424 (13)	0.0409 (12)	0.0012 (9)	0.0053 (9)	0.0008 (10)
C6	0.0402 (12)	0.0492 (14)	0.0346 (11)	0.0048 (11)	0.0043 (9)	0.0049 (11)
C7	0.0427 (12)	0.0580 (15)	0.0343 (11)	0.0027 (11)	0.0140 (10)	0.0054 (11)
C8	0.0314 (11)	0.0615 (16)	0.0363 (11)	0.0035 (11)	0.0129 (9)	0.0020 (11)
C9	0.0265 (10)	0.0340 (11)	0.0314 (10)	−0.0030 (9)	0.0068 (8)	−0.0043 (9)
C10	0.0263 (10)	0.0402 (12)	0.0284 (10)	−0.0014 (9)	0.0045 (8)	−0.0028 (9)
O11	0.0303 (8)	0.0941 (15)	0.0432 (9)	0.0096 (9)	0.0185 (7)	0.0201 (9)
O12	0.0274 (8)	0.0902 (14)	0.0523 (10)	0.0100 (9)	0.0169 (7)	0.0174 (10)
C13	0.0280 (10)	0.0351 (11)	0.0292 (10)	−0.0022 (9)	0.0081 (8)	−0.0043 (9)
C14	0.0331 (11)	0.0515 (14)	0.0342 (11)	−0.0001 (10)	0.0122 (9)	0.0077 (10)
C15	0.0267 (10)	0.0566 (15)	0.0429 (12)	−0.0002 (10)	0.0090 (9)	0.0054 (11)
C16	0.0327 (11)	0.0424 (13)	0.0320 (11)	0.0020 (10)	0.0010 (9)	0.0002 (10)
C17	0.0392 (12)	0.0537 (15)	0.0302 (11)	−0.0035 (11)	0.0109 (9)	0.0040 (10)
C18	0.0287 (10)	0.0497 (13)	0.0328 (11)	−0.0027 (10)	0.0100 (8)	0.0006 (10)
F19	0.0386 (7)	0.0798 (11)	0.0443 (8)	0.0069 (7)	−0.0013 (6)	0.0139 (7)

### Geometric parameters (Å, °)

**Table:**

O1—C10	1.363 (2)	C8—C10	1.388 (3)
O1—C2	1.374 (2)	C8—H8	0.9300
C2—C3	1.355 (3)	C9—C10	1.384 (3)
C2—C13	1.468 (3)	O11—H11	0.83 (3)
C3—O11	1.352 (2)	C13—C18	1.392 (3)
C3—C4	1.449 (3)	C13—C14	1.397 (3)
C4—O12	1.237 (2)	C14—C15	1.379 (3)
C4—C9	1.454 (3)	C14—H14	0.9300
C5—C6	1.367 (3)	C15—C16	1.360 (3)
C5—C9	1.403 (3)	C15—H15	0.9300
C5—H5	0.9300	C16—F19	1.358 (2)
C6—C7	1.392 (3)	C16—C17	1.368 (3)
C6—H6	0.9300	C17—C18	1.378 (3)
C7—C8	1.369 (3)	C17—H17	0.9300
C7—H7	0.9300	C18—H18	0.9300
			
C10—O1—C2	121.01 (15)	C5—C9—C4	122.85 (18)
C3—C2—O1	120.12 (18)	O1—C10—C9	122.00 (17)
C3—C2—C13	129.15 (18)	O1—C10—C8	116.41 (17)
O1—C2—C13	110.73 (16)	C9—C10—C8	121.58 (19)
O11—C3—C2	120.13 (19)	C3—O11—H11	109 (2)
O11—C3—C4	117.82 (17)	C18—C13—C14	117.60 (18)
C2—C3—C4	122.04 (18)	C18—C13—C2	123.34 (17)
O12—C4—C3	121.08 (19)	C14—C13—C2	119.05 (18)
O12—C4—C9	123.13 (19)	C15—C14—C13	121.43 (19)
C3—C4—C9	115.79 (17)	C15—C14—H14	119.3
C6—C5—C9	120.73 (19)	C13—C14—H14	119.3
C6—C5—H5	119.6	C16—C15—C14	118.73 (19)
C9—C5—H5	119.6	C16—C15—H15	120.6
C5—C6—C7	119.7 (2)	C14—C15—H15	120.6
C5—C6—H6	120.1	F19—C16—C15	118.55 (19)
C7—C6—H6	120.1	F19—C16—C17	119.32 (19)
C8—C7—C6	120.9 (2)	C15—C16—C17	122.1 (2)
C8—C7—H7	119.5	C16—C17—C18	119.08 (19)
C6—C7—H7	119.5	C16—C17—H17	120.5
C7—C8—C10	118.86 (19)	C18—C17—H17	120.5
C7—C8—H8	120.6	C17—C18—C13	121.04 (18)
C10—C8—H8	120.6	C17—C18—H18	119.5
C10—C9—C5	118.14 (18)	C13—C18—H18	119.5
C10—C9—C4	119.01 (18)		
			
C10—O1—C2—C3	1.5 (3)	C5—C9—C10—O1	179.34 (19)
C10—O1—C2—C13	−177.75 (18)	C4—C9—C10—O1	−1.9 (3)
O1—C2—C3—O11	−179.95 (19)	C5—C9—C10—C8	−1.0 (3)
C13—C2—C3—O11	−0.8 (4)	C4—C9—C10—C8	177.8 (2)
O1—C2—C3—C4	−1.4 (3)	C7—C8—C10—O1	179.6 (2)
C13—C2—C3—C4	177.7 (2)	C7—C8—C10—C9	0.0 (4)
O11—C3—C4—O12	−1.9 (3)	C3—C2—C13—C18	11.1 (4)
C2—C3—C4—O12	179.5 (2)	O1—C2—C13—C18	−169.68 (19)
O11—C3—C4—C9	178.3 (2)	C3—C2—C13—C14	−167.8 (2)
C2—C3—C4—C9	−0.3 (3)	O1—C2—C13—C14	11.4 (3)
C9—C5—C6—C7	−1.3 (4)	C18—C13—C14—C15	−0.4 (3)
C5—C6—C7—C8	0.2 (4)	C2—C13—C14—C15	178.6 (2)
C6—C7—C8—C10	0.4 (4)	C13—C14—C15—C16	0.0 (4)
C6—C5—C9—C10	1.7 (3)	C14—C15—C16—F19	179.7 (2)
C6—C5—C9—C4	−177.0 (2)	C14—C15—C16—C17	0.4 (4)
O12—C4—C9—C10	−177.9 (2)	F19—C16—C17—C18	−179.6 (2)
C3—C4—C9—C10	1.9 (3)	C15—C16—C17—C18	−0.3 (4)
O12—C4—C9—C5	0.8 (4)	C16—C17—C18—C13	−0.1 (4)
C3—C4—C9—C5	−179.4 (2)	C14—C13—C18—C17	0.5 (3)
C2—O1—C10—C9	0.2 (3)	C2—C13—C18—C17	−178.5 (2)
C2—O1—C10—C8	−179.50 (19)		

### Hydrogen-bond geometry (Å, °)

**Table:**

*D*—H···*A*	*D*—H	H···*A*	*D*···*A*	*D*—H···*A*
O11—H11···O12	0.83 (3)	2.28 (3)	2.722 (2)	113 (3)
O11—H11···O12^i^	0.83 (3)	2.02 (3)	2.761 (2)	149 (3)

Symmetry codes:  (i) −*x*+1, −*y*+2, −*z*+1.

### Table 2 C—F···π interactions (Å, °).

*Cg*1 is the centroid of the C13–C18 ring.

**Table:**

*X*	*I*	*J*	*I···J*	*X···J*	*X—I···J*
C16	F19	*Cg*1^ii^	3.888 (2)	3.642 (2)	69.5 (2)

Symmetry code: (ii)  x, y – 1, z.

## References

[bb1] Aakeröy, C. B., Seddon, K. R. & Leslie, M. (1992). *Struct. Chem.* **3**, 63–65.

[bb2] Cantrell, J. S. & Stalzer, R. A. (1982). *Acta Cryst.* B**38**, 983–984.

[bb3] Demchenko, A. P., Klymchenko, A. S., Pivovarenko, V. G. & Ercelen, S. (2002). *Fluorescence Spectroscopy, Imaging and Probes – New Tools in Chemical, Physical and Life Sciences*, edited by R. Kraayenhof, A. J. W. G. Viser & H. C. Gerritsen, Vol. 2 (Springer Series on Fluorescence), pp. 101–110. Berlin, Heidelberg: Springer-Verlag.

[bb4] Dorn, T., Janiak, C. & Abu-Shandi, K. (2005). *CrystEngComm*, **7**, 633–641.

[bb5] Etter, M. C., Urbańczyk-Lipkowska, Z., Baer, S. & Barbara, P. F. (1986). *J. Mol. Struct.* **144**, 155–167.

[bb6] Farrugia, L. J. (1997). *J. Appl. Cryst.* **30**, 565.

[bb7] Oxford Diffraction (2008). *CrysAlis CCD* and *CrysAlis RED* Oxford Diffraction Ltd, Yarnton, England.

[bb8] Pivovarenko, V. G., Wróblewska, A. & Błażejowski, J. (2005). *Anal. Chim. Acta*, **545**, 74–78.

[bb9] Roshal, A. D., Moroz, V. I., Pivovarenko, V. G., Wróblewska, A. & Błażejowski, J. (2003). *J. Org. Chem.* **68**, 5860–5869. 10.1021/jo034200f 12868918

[bb10] Sengupta, P. K. & Kasha, M. (1979). *Chem. Phys. Lett.* **68**, 382–385.

[bb11] Sheldrick, G. M. (2008). *Acta Cryst.* A**64**, 112–122. 10.1107/S0108767307043930 18156677

[bb12] Smith, M. A., Neumann, R. M. & Webb, R. A. (1968). *J. Heterocycl. Chem.* **5**, 425–426.

[bb13] Spek, A. L. (2009). *Acta Cryst.* D**65**, 148–155. 10.1107/S090744490804362X PMC2631630 19171970

[bb14] Waller, M. P., Hibbs, D. E., Overgaard, J., Hanrahan, J. R. & Hambley, T. W. (2003). *Acta Cryst.* E**59**, o767–o768.

